# Molecular Memory Micromotors for Fast Snake Venom Toxin Dynamic Detection

**DOI:** 10.1021/acs.analchem.4c01976

**Published:** 2024-06-24

**Authors:** Javier Bujalance-Fernández, Beatriz Jurado-Sánchez, Alberto Escarpa

**Affiliations:** † Department of Analytical Chemistry, Physical Chemistry, and Chemical Engineering, Universidad de Alcala, Alcala de Henares, E-28805 Madrid, Spain; ‡ Chemical Research Institute “Andres M. del Rio”, Universidad de Alcala, E-28805 Madrid, Spain

## Abstract

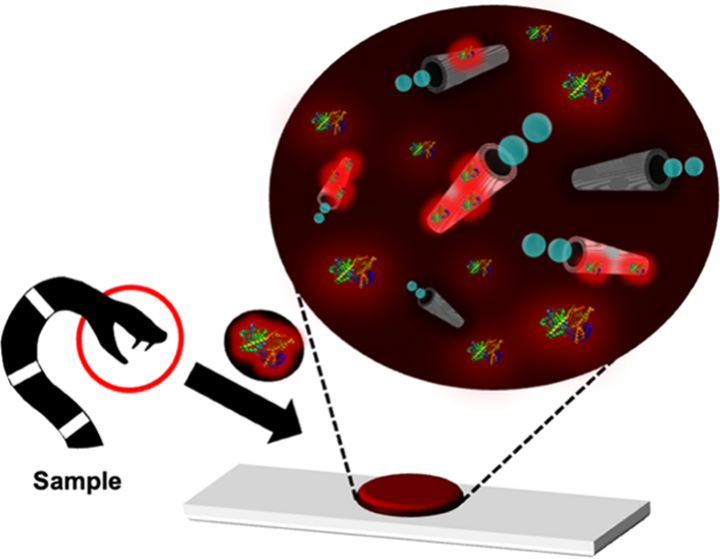

The analysis and detection of snake venom toxins are a matter of great importance in clinical diagnosis for fast treatment and the discovery of new pharmaceutical products. Current detection methods have high associated costs and require the use of sophisticated bioreceptors, which in some cases are difficult to obtain. Herein, we report the synthesis of template-based molecularly imprinted micromotors for dynamic detection of α-bungarotoxin as a model toxin present in the venom of many-banded krait (*Bungarus multicinctus*). The specific recognition sites are built-in in the micromotors by incubation of the membrane template with the target toxin, followed by a controlled electrodeposition of a poly(3,4-ethylenedioxythiophene)/poly(sodium 4-styrenesulfonate) polymeric layer, a magnetic Ni layer to promote magnetic guidance and facilitate washing steps, and a Pt layer for autonomous propulsion in the presence of hydrogen peroxide. The enhanced fluid mixing and autonomous propulsion increase the likelihood of interactions with the target analyte as compared with static counterparts, retaining the tetramethylrhodamine-labeled α-bungarotoxin on the micromotor surface with extremely fast dynamic sensor response (after just 20 s navigation) in only 3 μL of water, urine, or serum samples. The sensitivity achieved meets the clinically relevant concentration postsnakebite (from 0.1 to 100 μg/mL), illustrating the feasibility of the approach for practical applications. The selectivity of the protocol is very high, as illustrated by the absence of fluorescence in the micromotor surface in the presence of α-cobratoxin as a representative toxin with a size and structure similar to those of α-bungarotoxin. Recoveries higher than 95% are obtained in the analysis of urine- and serum-fortified samples. The new strategy holds considerable promise for fast, inexpensive, and even onsite detection of several toxins using multiple molecularly imprinted micromotors with tailored recognition abilities.

## Introduction

Snake venoms consist of a mixture of up to 100 components, mostly peptide and protein toxins, with cytotoxic, neurotoxic, or hemotoxic effects, among others.^1,2^ The development of fast, easy, and cost-effective methods for the detection of such analytes is crucial, first due to snake bites can be lethal, leading to approximately 140,000 deaths worldwide.^3^ Prompt and accurate identification is of paramount significance for fast treatment. Second, snake venom toxins are also explored for the development of new pharmaceuticals or as biological markers to understand certain biological processes.^4^ Third, forensic laboratories are facing the diagnosis of accidental deaths due to venom abuse and addiction.^5^ Several methods have been developed for snake venom detection, including immunoassays^6−9^ and DNA^10^-based assays or mass spectrometry approaches.^11^ Yet, these methods have a high associated cost and require a long analysis time (>2 h).^12^

Recent trends in snake venom toxin analysis are aimed at the design of point-of-care tools for ultrafast detection. Lateral flow assays (LFAs) meet such a demand. Such tools are normally based on the use of specifically designed antibodies toward targeted venoms from different species, facilitating optical detection with portable smartphones in just 15 min. LFAs also provided a limit of detection that met the clinically relevant range postsnakebite (from 0.1 to 1000 ng/mL).^13−18^ It should be mentioned here, yet, the performance can be affected by the matrix influence when applied to blood or serum samples.^19^ Paper-based optical assays have also been developed, using antibodies in connection with the enzyme horseradish peroxidase and the colorimetric substrate tetramethylbenzidine for β-bungarotoxin detection in blood, plasma, and urine. The limits of detection were within the range of the LFA, with a detection time of 25 min.^18^ Specific aptamers designed by SELEX technology have been used for the detection of β-bungarotoxin and other components from the venom of *B. caeruleus* in a paper-based colorimetric assay format.^20^ While useful and fast, all of the above-mentioned methods require the use of specific antibodies or specifically designed aptamers, which are somewhat expensive and limit the application of specific toxins and components. As an alternative, a fluorescence-based assay was constructed by using eosin Y, fluorescein isothiocyanate isomer I (FITC), sulforhodamine B, and titan yellow fluorescent dyes, which present specific affinity for the detection of phospholipase A2, α-cobratoxin (α-CT), cardiotoxin, hyaluronidase, thrombin, and hemocoagulase as representative components of snake venom. Electrostatic and noncovalent interactions of the charged target compounds with the specific dyes result in fluorescence quenching in a concentration-dependent manner. The array was able to detect and discriminate the toxins at a concentration of 1 mg/mL in 2 min.^21^

Inspired by the current analytical needs for snake venom toxin detection and given the current developments in this direction toward fast, cheap, and (multiplexed) onsite analysis, herein, we report on a molecularly imprinted micromotor (MIP-MM)-based approach for the determination of α-bungarotoxin (α-BTx) as proof-of-concept toxin from *Bungarus multicinctus*. Molecularly imprinted polymers (MIPs), also known as plastic antibodies, are particularly attractive for fluorescence-based detection approaches with high selectivity in biosensors. Tailored binding sites are introduced by polymerization of a given monomer in the presence of the target analyte. The resulting polymers have specific recognition affinity but compared to antibodies, they offer improved stability and greatly reduced costs. Most importantly, such technology possesses high versatility along with the ability to perform multiplexed analysis.^22,23^

On the other hand, MMs are microscale devices that can propel autonomously in solutions, thus holding considerable promise in analytical sensing.^24−26^ Particularly, catalytic MMs propelled by the decomposition of hydrogen peroxide in catalyst layers generate an enhanced fluid mixing that allows operation in microliter sample volumes, greatly reducing the analysis time.^27^ The template electrosynthesis approach for the preparation of tubular MMs allows us to prepare a myriad of designs with different outer layers composed of carbon and 2D nanomaterials,^28−30^ polymers,^29,31,32^ etc., with an inner catalytic layer (normally Pt). The rich chemistry of the outer layer has been exploited for functionalization with DNA,^33,34^ antibodies,^35,36^ aptamers,^37,38^ or affinity peptides^39^ for the detection of clinically relevant biomarkers.

MIP technology can be easily combined with MMs by using the template electrosynthesis route. To this end, the membrane template used to obtain the tubular structures is previously incubated with the analyte, followed by electrodeposition of a polymeric layer. After cleaning and removal of the analyte, the resulting MMs display tailored recognition sites for specific isolation and detection. The first tubular MIP-MMs were prepared by electropolymerization of poly(3,4-ethylenedioxythiophene) (PEDOT) in the presence of FITC-labeled avidin as the target analyte. The MMs were able to capture the fluorescence-labeled analyte efficiently in serum and saliva.^40^ Later on, the same strategy was adopted for the synthesis of PEDOT/Ni/Pt MMs for phycocyanin detection. Such an analyte is a native fluorescence protein associated with the presence of cyanobacteria in the environment. Detection of the target analyte was achieved at concentrations of up to 1 mg/mL, with efficient operation in seawater. While promising, the full analytical performance of MIP-MM-based detection strategies remains largely unexplored toward realistic applications.^41^ In the environmental field, light-driven BiVO_4_ MMs have been modified with MIP sites to remove undesired contaminants and improve the efficiency of the pollutant removal process.^42^ Herein, we report the synthesis of α-BTx MIP-MMs by electropolymerization of PEDOT in the presence of the tetramethylrhodamine (TRITC)-labeled analyte. The resulting MMs have targeted recognition sites toward the analyte, with increasing fluorescence intensity on the surface in a concentration-dependent manner. The MMs possess an intermediate magnetic Ni layer and a catalytic Pt layer for efficient propulsion in the presence of hydrogen peroxide. The enhanced fluid mixing and autonomous propulsion enhanced the likelihood of interaction with the target analyte compared with static counterparts. In the following sections, we will illustrate the synthesis and characterization of the MIP-MMs as well as the influence of the surfactant in efficient propulsion and interaction with the analyte. Unlike previous works, the analytical performance will be characterized through the study of the main analytical characteristics in complex media, such as serum and urine. The selectivity of the strategy will be tested in the presence of α-CT as another representative venom toxin of some species of snakes of the genus *Naja*, which cohabit in the same habitats. The strategy holds considerable promise for fast and cheap detection of a myriad of venom-associated toxins even in multiplexed and with enormous potential for onsite assays using specific MIP-MMs synthesized in the presence of different target analytes.

## Materials and Methods

### Reagents and Materials

Cyclopore polycarbonate (PC) membranes (5 μm pores, 25 mm diameter, cat. WHA70602513), α-BTx-TRITC (cat. T0195-5MG), poly(sodium 4-styrenesulfonate) (PSS) (cat. 243051), 3,4-ethylenedioxythiophene (EDOT) (cat. 483028), nickel(II) sulfamate tetrahydrate (cat. 262277), nickel(II) chloride hexahydrate (cat. N6136), chloroplatinic acid solution (cat. 262587), 2-propanol (cat. 278475), sodium dodecyl sulfate (SDS) (cat. 71727), poly(ethylene glycol) (PEG) (cat. 89510), rhodamine 6G (cat. R4127), quinine hydrochloride dihydrate (cat. 8.22194), and human serum (cat. H4522) were purchased from Sigma-Aldrich (Spain). Human urine (cat. U2500-09-1L) and recombinant *Naja kaouthia* α-CT labeled with FITC (cat. CUST-CAT-12052023-2A) were purchased from Quimigen. Dichloromethane (cat. no. 34856) and fluorescein (cat. no. 32615) were purchased from Honeywell (Spain). Ethanol absolute (cat. ET0005005P) was provided by Scharlau (Spain). Hydrogen peroxide 30% (v/v) (cat. HYPE-30P-1K0) was purchased from Labbox. Boric acid (cat. 180570010) and hydrochloric acid (cat. 10000180) were acquired from Fisher Scientific. All of the reagents were used without further purification. Milli-Q water was obtained using a Millipak Express Filter (cat. MPGP04001), and a Vent Filter (cat. TANKMPK01) was purchased from Merck Millipore (Germany).

### Equipment

An ultrasonic bath (Elmasonic S 30 H) was used to clean the membranes and the MMs. A potentiostat Autolab PGSTAT 12 (Eco Chemie, Utrecht, Netherlands) was used for the electrodeposition of the MMs. An inverted Nikon Eclipse Instrument Inc. Ti-S/L100 optical microscope coupled with a Zyla sCMOS camera was used to capture images. The microscope is equipped with a xenon arc lamp light source system (Sutter instrument company, LB-LS/30) attached and DAPI-5060C (λ_ex_ 377/50 nm), FITC (λ_ex_ 480/30 nm) and G-2A (λ_ex_ 535/50 nm) filter cubes (Nikon) to filter different wavelength to excite the molecules. The fluorescence of the MMs was analyzed using ImageJ software, and the videos and speed were recorded and measured using NIS-elements software. Scanning electron microscopy (SEM) characterization of the MMs was performed using a JEOL JSM 6335F microscope (JEOL USA, Massachusetts) coupled to an energy-dispersive X-ray (EDX) system (Xflash detector 4010 (Bruker, Massachusetts)). An Eppendorf Centrifuge 5430 instrument attached with an FA-45-30-11 rotor was used to wash the MMs. Membrane gold sputtering was carried out at the “Centro de Apoyo a la Investigación″ Electronic Microscopy Service of the University of Alcala. A Zetasizer Nano ZS (Malvern Panalytical, United Kingdom) was used to measure the zeta potential, and the data was analyzed using Malvern Zetasizer software. Measurements were performed at 25 °C, with an index refraction of 1.6, an absorption of 0.01, and at pH 7. Origin 8.5 software was used to generate graphs of the results.

### MIP-MM and Control MM Synthesis

**Scheme 1:**
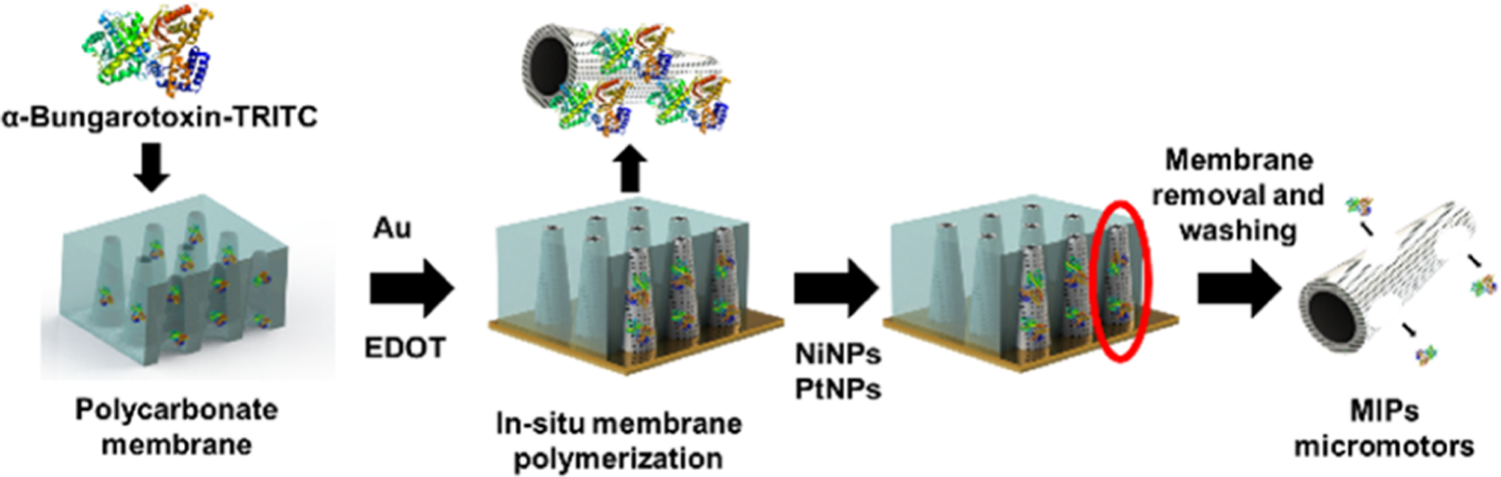
Schematic of the Synthesis of the MIP-MMs for α-BTx-TRITC Detection

The schematic of the synthesis is illustrated in Scheme 1. In the first step, the PC membrane was sonicated in an ultrasonic bath inside a 2 mL microtube with water for 3 min to remove air and some possible impurities from its pores. Next, the membrane was incubated with a solution containing 0.05 mg/mL α-BTx for 1 h at room temperature under shaking conditions, followed by two washes with water to remove any external protein residues. The incubated PC membrane was then sputtered with a ∼50 nm gold layer to serve as a working electrode in the electrochemical cell. The same procedure was carried out on membranes for the synthesis of non-MIP-MMs (control), except for the incubation, which was done with ultrapure water. In the second step, the PC membrane was assembled in the electrodeposition cell, following electropolymerization of a PEDOT layer from a plating solution containing 10 mM EDOT and 270 mM PSS, at +0.80 V using a charge of 2C. The intermediate Ni layer was electrodeposited by galvanostatic voltammetry in two different steps: 10 pulses of 0.1 s (−20 mA) and one deposition scan of 300 s (−6 mA), from a solution containing 0.91 M nickel sulfamate, 82 mM nickel chloride, and 0.48 M boric acid at pH 4. The catalytic Pt layer was electrodeposited amperometrically for 750 s at −0.4 V from a solution containing 4 mM chloroplatinic acid and 175 mM boric acid. In all cases, Ag/AgCl was used as the reference electrode, and a Pt wire was used as the counter electrode. In the third step, the PC membrane was removed from the cell, polished with an alumina slurry to remove the gold layer, and dissolved in dichloromethane (2 times, 30 min), followed by MM dispersion in ethanol, isopropanol, and water.

To remove the α-BTx contained within the imprinted polymer, micromotors were washed and shaken in a 0.5 mL microtube with SDS 5% (w/v) for 3 min, washed and shaken again with SDS 10%, and washed twice with water. The same treatment was carried out to control the MMs.

### MIP-MM and Control MM Speed Characterization

The speed of the MMs was tracked by placing 1 μL of MM solution, 1 μL of hydrogen peroxide, 1 μL of the surfactant, and 1 μL of water, serum, or urine on a glass slide on top of the optical microscope. For experiments in urine and serum, the surfactant solutions were prepared in this medium to minimize dilutions. Videos were taken using the 20× objective at 40 frames per second. The speed was analyzed with NIS-tracking software.

### Micromotor Counting

Detection experiments were performed at a fixed concentration of 500,000 MMs/mL. To achieve this ratio, a sample of 1 μL was photographed at 4× objective and then photographed again 4 times at 20× objective in different parts of the drop. The area of the 1 μL drop was measured, and the number of MMs per each 20× image (with a known area) was counted, allowing the average number of MMs per area and therefore per μL to be calculated. This technique was performed 4 times per batch of MMs. Finally, they were resuspended in a volume of 10% (w/v) SDS or PEG calculated to obtain the desired concentration to be kept ready for subsequent experiments. This synthesis method can produce approximately 750,000 MMs per batch.

### α-BTx-TRITC Detection with the MMs

Response time was optimized by taking images at different times (from 0 to 600 s) of the control and MIP-MMs in a solution containing 10% hydrogen peroxide, 3.3% PEG, and 6 μg/mL α-BTx-TRITC, with 20× objective, 100 ms exposure time, a G-2A filter cube, and a xenon arc lamp light source. Calibration curves were performed in serum (20%, MIP-MMs), urine (20%, MIP-MMs), and water (MIP-MMs and control MMs) with 4 different concentrations of α-BTx-TRITC (0, 5, 10, and 15 μg/mL), using the same conditions above-described. Images were taken at times 0 and 20 s. Final values were obtained using ImageJ software by subtracting the fluorescence of the 0 s values from the 20 s values. Selectivity experiments were performed in water. Rhodamine 6G (0.15 μg/mL) was measured with a G-2A filter, fluorescein (60 μg/mL) dissolved in EtOH/H_2_O (1:10) was measured with a DAPI-5060C filter, quinine hydrochloride (60 μg/mL) dissolved in HCl 0.15 M was measured with a DAPI-5060C filter, and α-CT-FITC (15 μg/mL) was measured with a C-FL-C FITC filter individually. In the case of α-CT-FITC, the selectivity was also evaluated in serum (20%), urine (20%), and water.

Please note that to facilitate visual identification, the color of the images shown in this paper has been changed from a grayscale (original image) to an RGB scale. Also, the contrast and brightness of some images have been changed from those of the original to facilitate visual identification. All measures of fluorescence intensity in this paper were taken from the original image and not from the processed images.

## Results and Discussion

**Figure 1:**
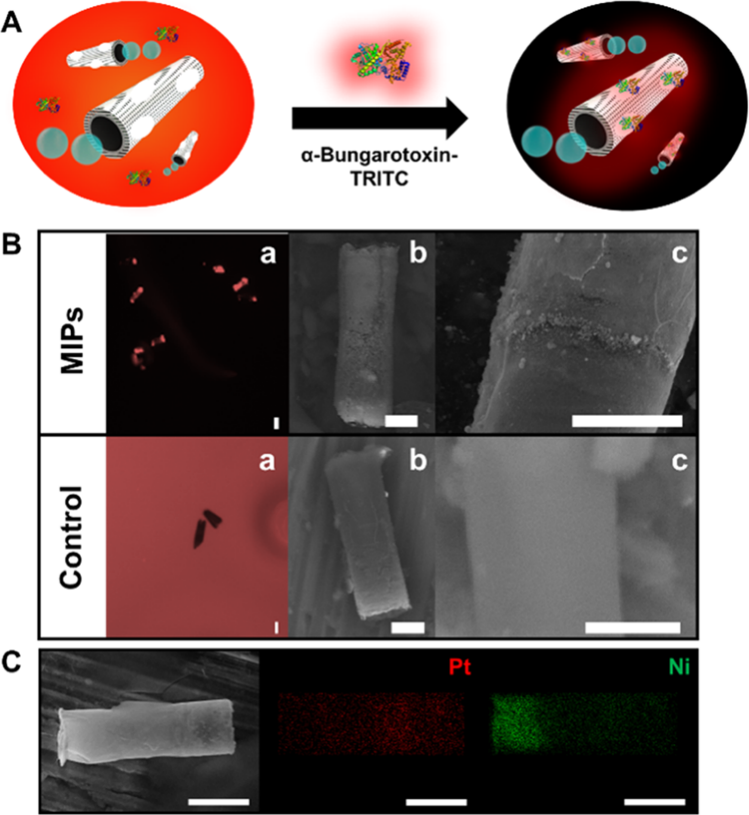
(A) Schematic of the detection of α-BTx-TRITC using MIP-MMs. Change in fluorescence distribution from ON (solution)/OFF (MMs) to OFF (solution)/ON (MMs). (B) Time-lapse microscopy images of α-BTx-TRITC solutions (15 μg/mL) after 20 navigated MIP-MMs and PEDOT control MMs (a) and SEM images showing the morphology of the MMs (b, c). Scale bars: 2.5 μm. (C) SEM and EDX images illustrating the morphology and element distribution of the MMs. Scale bars, 5 μm. Conditions: 3.3% PEG, 10% H_2_O_2_.

Figure 1A illustrates the schematic of the MIP-MM-based fluorescence approach for α-BTx-TRITC detection. The tailored MMs navigate in solutions containing increasing concentrations of the target toxin, which is retained in the specific molecular sites, increasing the fluorescence on the surface of the MMs in a concentration-dependent manner. Such an analyte was selected as a proof-of-concept toxin with good commercial availability and biosafety features for manipulation in the laboratory with standard safety measurements. α-BTx is a neurotoxin present in the venom of *Bungarus multicintus*. It represents the main fraction present, along with the β and γ analogs. α-BTx structure is a single polypeptide chain of 74 amino acids (molecular weight, 8.01 kDa) cross-linked by five disulfide bridges.^43^ To facilitate observation and quantification, in this work, we used the analyte labeled with fluorescence TRITC.^44^ Please note here that the sample can be easily labeled with the commercially available kits, or the analyte can be used in connection with the nonlabeled analogous form (both forms are commercially available) in a competitive assay format. The labeled target toxin has a positive charge^45^ at neutral pH, while the PC membrane is negatively charged,^46^ promoting the interaction via electrostatic interactions. Next, EDOT was chosen as a polymer for PEDOT electropolymerization due to its ideal characteristics for the preparation of MIP-MMs.^40,41^ PSS, a negatively charged polymer, was used in connection with EDOT to improve its solubility and dispersion in aqueous solutions,^47^ improving the yield of synthesis and resulting in reproducible MIP-MMs. Next, the magnetic Ni layer (to promote magnetic guidance and facilitate washing steps) and the catalytic Pt layer were electrodeposited and the MMs were released, as described in the Materials and Methods Section. The resulting MMs have built-in recognition capabilities for the target α-BTx-TRITC. To check the successful generation of the MIP recognition sites, the MMs were placed to navigate in a solution containing 15 μg/mL α-BTx-TRITC. Control PEDOT MMs were also used similarly. The exposure time was set at 100 ms using the Xe arc lamp as an excitation source and the G-2A filter cube. Videos were taken, and the fluorescence of the MMs was measured with ImageJ at 0 and 20 s (see the Materials and Methods section for more details). As can be seen in Figure 1B, the MIP-MMs are covered with red fluorescence from the labeled toxin, while no apparent fluorescence is observed on the surface of PEDOT control MMs, illustrating the successful built-in recognition abilities of our MMs.

Further, SEM characterization (see Figure 1B, b and c) illustrates the presence of rough and patchy-like surfaces on the MIP-MMs (which can be attributed to the recognition sizes) as compared with the smooth morphology of the control PEDOT MMs. The EDX images of Figure 1C reveal the composition of the inner Ni and Pt layers evenly distributed along the microtubes, which have an average diameter of 5 μm and an average length of 12 μm.

**Figure 2:**
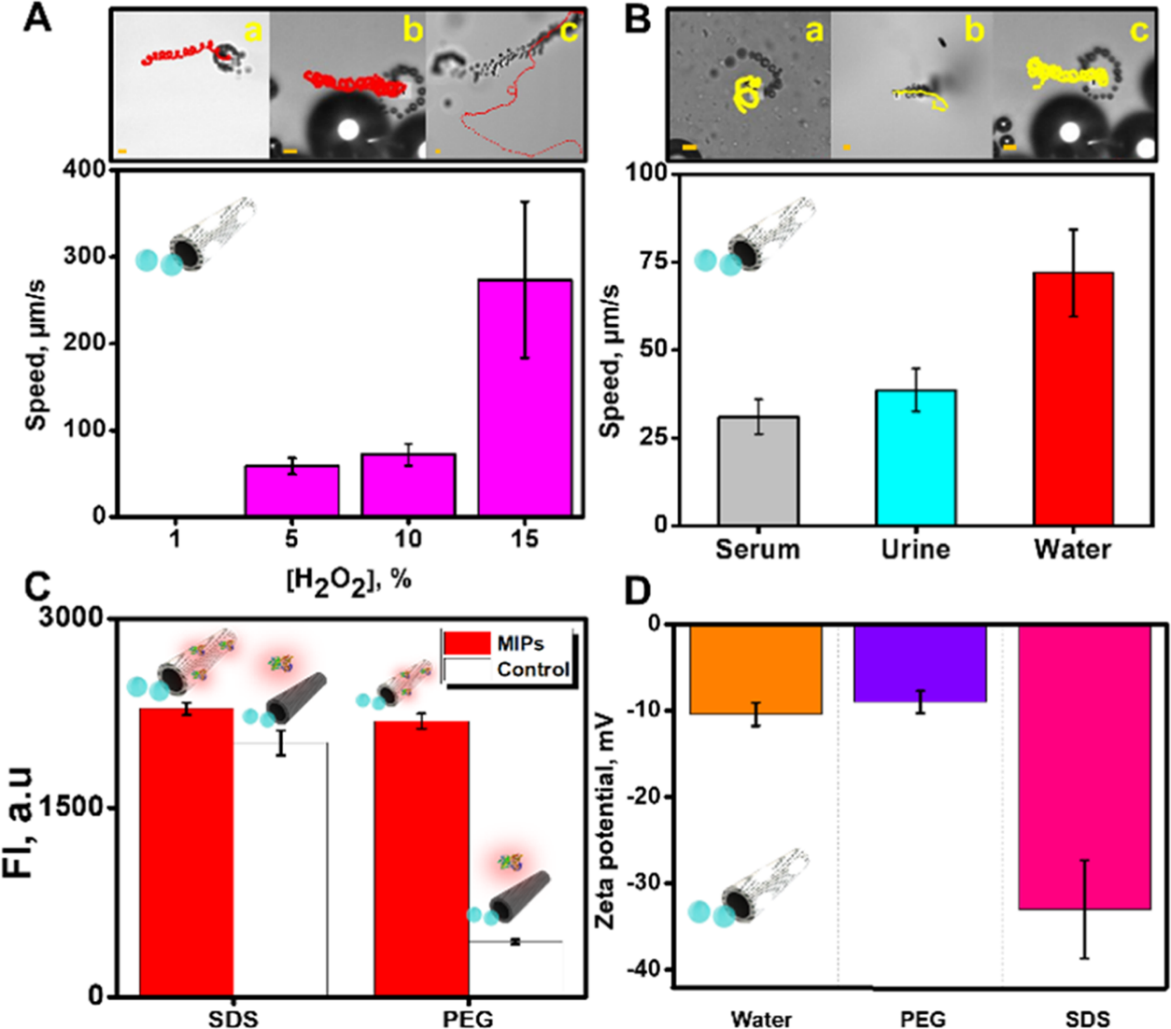
Characterization of the MIP-MM propulsion. (A) Time-lapse images (taken from Video S1) of the propulsion of MIP-MMs at 5 (a), 10 (b), and 15% (c) H_2_O_2_ using 3.3% PEG as the surfactant and corresponding speed plot (bottom). (B) Time-lapse images (taken from Video S2) of the propulsion of the MIP-MMs in serum (a), urine (b), or water samples (c) using 10% H_2_O_2_ as fuel and 3.3% PEG as the surfactant and corresponding speed plots. (C) Influence of the surfactant (3.3%) in the detection of α-BTx-TRITC (in terms of fluorescence intensity, 15 μg/mL) with the MIP-MMs using 10% H_2_O_2_ as fuel. Fluorescence values were plotted by subtracting the fluorescence values at time 0 from those at 20 s. (D) Z-potential values (mV) in micromotors in water, with 3.3% PEG and with 3.3% SDS. Scale bars: 10 μm. Error bars correspond to the standard deviation of 5 (from A to C) or 3 (D) measurements.

Once we tested the successful synthesis of the MMs and before further evaluating the analytical performance, we studied the propulsion in different media and the potential influence of the surfactant and media constituents on the detection and potential nonspecific interactions. PEG was initially chosen as a surfactant due to its biocompatibility to avoid potential α-BTx-TRITC denaturalization and neutral charge to avoid unspecific, electrostatic interactions.^48^ As can be seen in Figure 2A and Video S1, the speed of the MMs increases along with the concentration of H_2_O_2_.^49^ As a compromise among a higher number of motile MMs and the lowest amount of hydrogen peroxide for propulsion, we choose 10% H_2_O_2_ levels at optimal. The number of MMs that moved at concentrations of 10 and 15% was the total number of MMs. At such a level, almost 95% of the MMs move. Yet, we did not perform profound studies on this, as it is not considered crucial for the sensing procedure. The possible effect of nonmotile micromotors was eliminated by measuring the fluorescence intensity of 12 motile MMs in each measurement. We next tested the propulsion of the MMs in serum and urine samples, where the presence of proteins can bind to the Pt layer, reducing the catalytic activity, and the relatively high viscosity of the media can reduce the drag force of the MMs.^50,51^ As can be seen in Figure 2B, the speed decreases from 72 ± 12 μm/s in water to 39 ± 6 μm/s (urine) and 31 ± 6 μm/s (serum). Similar results were obtained for the PEDOT control MMs. Such speed decreases, however, do not hamper the further practical applicability of MMs for detection.

After evaluating the successful MM propulsion, we tested the effect of the surfactant on the specific interaction with the labeled analyte. As can be seen in Figure 2C, the use of SDS, a highly negatively charged anionic surfactant, results in nonspecific adsorption/interaction of α-BTx-TRITC, as reflected by the high fluorescence intensity on the surface of both the MIP and control MMs. In contrast, when neutral PEG was used as a surfactant, the fluorescence increase was only noted on the MIP-MMs, with no fluorescence in the control PEDOT, revealing the absence of unspecific interactions. To gain further insights into such phenomena, Z-potential measurements of the MMs were performed after incubation in the different surfactants. After contact with water at neutral pH, a slightly negative potential of −10 mV was recorded due to the PSS present in the PEDOT layer of the MMs. A similar potential value was obtained after incubation with neutral PEG. Yet, after incubation with SDS, a highly negative potential of −35 mV was obtained. Similar values were obtained for the control PEDOT MMs. Such negative charge promotes electrostatic interactions with the positively charged α-BTx-TRITC, responsible for the nonspecific interactions with both the imprinted and nonimprinted sites.

**Figure 3:**
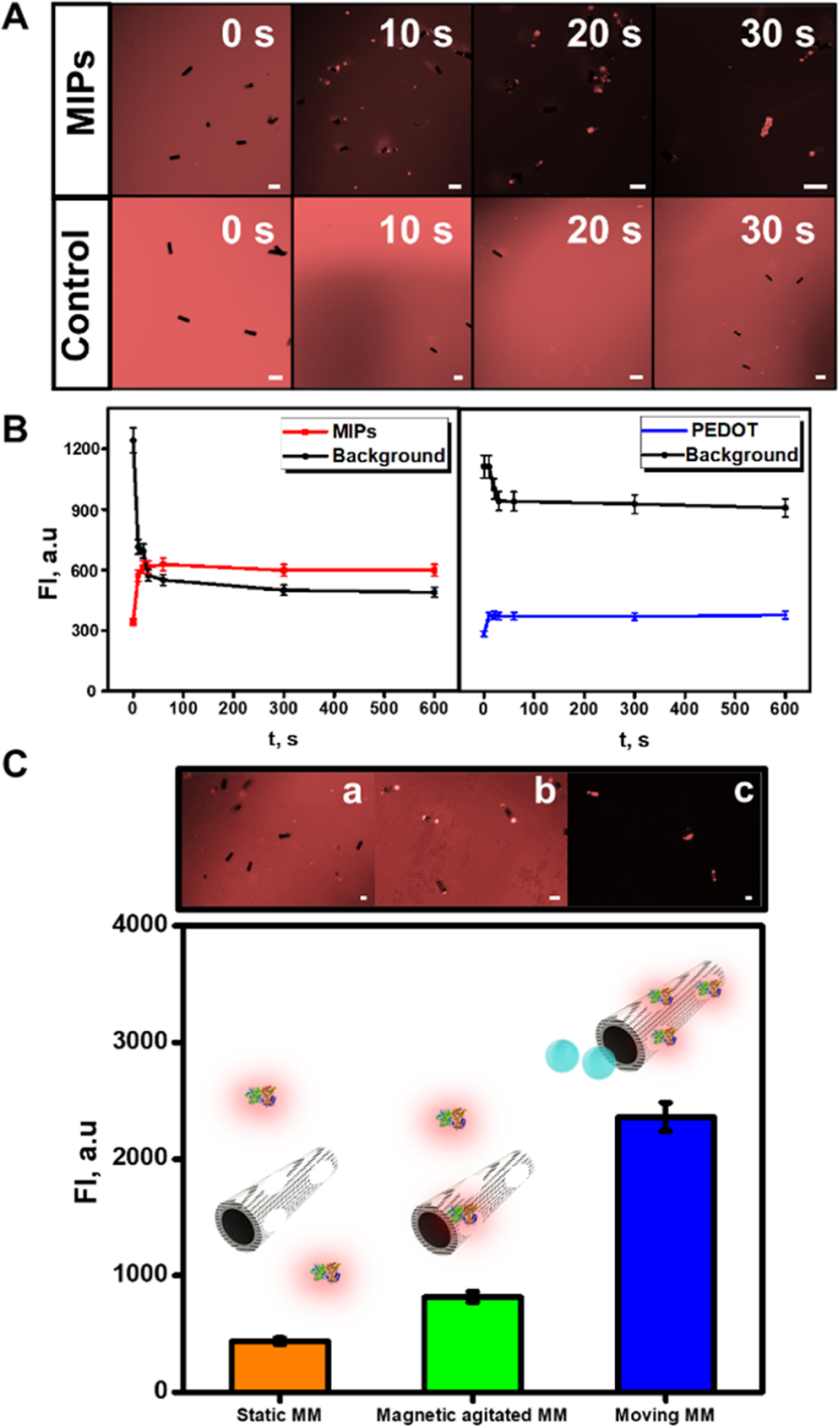
Response time of the MIP-MMs moving sensor for α-BTx-TRITC detection and the role of enhanced micromotor movement on detection. (A) Time-lapse fluorescence microscopy images taken after MIP-MMs and control PEDOT MM navigation in solutions containing 6 μg/mL α-BTx-TRITC at different times. (B) Corresponding graphics showing the FI values of the solutions (denoted as background) and the MIP-MMs and control PEDOT MMs. C) Influence of the enhanced MIP-MM movement on the detection: graphic showing the FI of the MIP-MMs in static (a), magnetic agitation (b), and moving in peroxide conditions (c) at 15 μg/mL α-BTx-TRITC levels. The top part shows the corresponding time-lapse fluorescence images at different conditions. Fluorescence values were plotted by subtracting the fluorescence values at time 0 from those at 20 s. Conditions: 3.3% PEG, 10% H_2_O_2_, response time (C), 20 s. Scale bars: 20 μm (A) and 10 μm (C). Error bars correspond to the standard deviation of 10 measurements.

After successful MM synthesis and optimization of the propulsion features and surfactant, the response time of the moving microsensors and the role of the enhanced fluid mixing in the detection were evaluated. Figure 3A and the corresponding plots of Figure 3B show fluorescence images over 10 s periods of solutions containing 6 μg/mL α-BTx-TRITC after MIP and control MM navigation for 60 s at optimal conditions.

The images and the plot reveal a clear increase in the fluoresce intensity (from 300 to 620 a.u.) and coverage on the MIP-MMs, with a subsequent decrease in the FI of the background (from 1200 to 600 a.u). The change was ultrafast, with a response time of the dynamic microsensors of 20 s and no changes after that time, as observed in the continuous line in the plot of Figure 3B. Please note that the line corresponding to the FI of the MIP-MMs surpasses that corresponding to the FI of the background. In the case of control PEDOT MMs, no apparent changes are noted in the fluorescence on the surface or the background over 60 s. Yet, in the corresponding plot, a slight increase in the fluorescence was noted (from 300 to 390 a.u.), with a subsequent decrease in the background (from 1190 to 920 a.u.), probably due to unspecific interaction of the slightly negatively charged MMs with the positively charged analyte. Please note that this effect is negligible compared with the high increase noted in the MIP-MMs. Next, we studied the effect of autonomous MM fluid mixing on the detection. As shown in Figure 3C, no changes in the fluorescence intensity of the MIP MMs on static conditions (no movement) are noted after incubation with solutions containing 15 μg/mL α-BTx-TRITC. With magnetic-agitated MMs (moving by a magnetic sitter), the fluorescence intensity increases to 900 a.u., much lower than the 2100 a.u. obtained with the catalytically propelled moving MMs. This revealed the capability of the self-propelled MMs to move in ultraminiaturized sample volumes (please note that here we used 3 μL of the sample), as compared with magnetic and other stirring procedures.^52^

**Figure 4:**
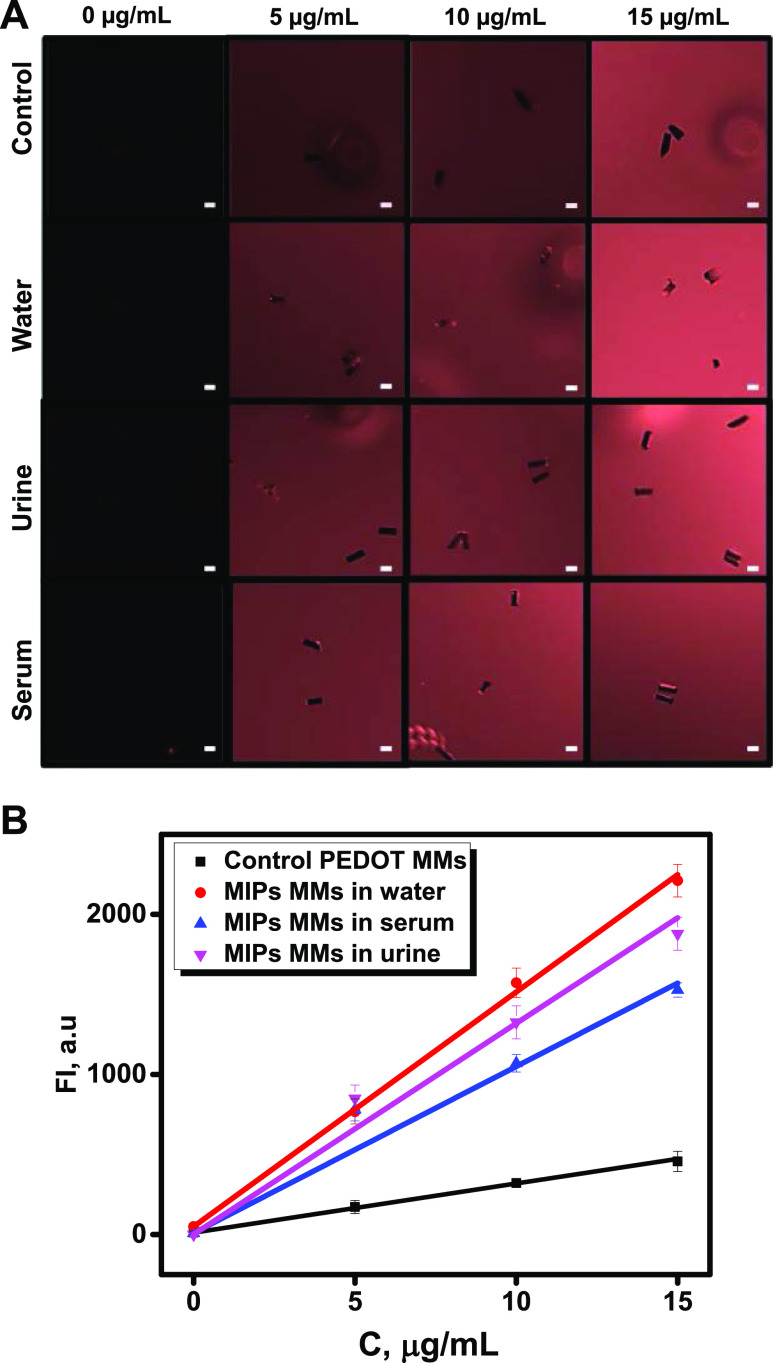
Analytical performance of MIP-MMs for α-BTx-TRITC detection. (A) Time-lapse fluorescence microscopy images corresponding to calibration plots in control experiments (with PEDOT MMs) and in ultrapure water, urine, and serum after 20 s of navigation in the fortified samples. (B) Corresponding calibration plots. Conditions: 3.3% PEG, 10% H_2_O_2_, response time, 20 s. Scale bars, 10 μm. Fluorescence values were plotted by subtracting the fluorescence values at time 0 from those at 20 s. Error bars represent the standard deviation of 12 measurements.

Under the optimized motion and detection conditions, calibration plots were conducted in ultrapure water, serum, and urine to check the potential matrix effects. Calibration was also conducted with the control PEDOT MMs without an imprint. Figure 4A shows time-lapse images after 20 navigates of the MMs in solutions containing increasing concentrations of the α-BTx-TRITC. For more details, please check the Materials and Methods section. The corresponding calibration plots are shown in Figure 4B. The images reflect the increasing fluorescence in the background because of the increase in the concentration of the marked analyte. Yet, it can be observed that as the concentration of toxin increases, the coverage and fluorescence surface in the MMs also increase. Please note that in control PEDOT MMs, no fluorescence increase in the MM surface is observed. The MMs can be retained with a magnet to remove the solution and redispersion in water, maximizing the fluorescence emission for better visualization. Yet, please note here that the ImageJ program can detect substantial changes in the fluorescence intensity, avoiding further steps that can delay detection. As can be seen in Figure 4B, linear calibration plots were obtained with a clear increase in the fluorescence intensity in the MM surface as the concentration of α-BTx-TRITC increased. Please note the much lower response in the case of PEDOT control MMs, which is mainly attributed to the nonspecific interactions previously described, which do not hamper detection. The main analytical characteristics are plotted in Table 1.

**Table 1:** Analytical Characteristics

	LR (μg/mL)	*r*	LOD (μg/mL)	slope (FI/μg mL^-1^)
water	0.7–15	0.999	0.2	147 ± 3
serum	1.2–15	0.996	0.4	104 ± 7
urine	1.0–15	0.995	0.3	132 ± 10

The linear range (LR) spans up to 15 μg/mL, with good correlation coefficients higher than 0.995. The limit of detection (LOD) and quantification (LOQ) were calculated as 3 or 10 times the error of the ordinate (obtained by plotting the calibration data with the origin) divided by the slope of the calibration plot.

To check the matrix effect, we calculated the confidence interval (95% probability) of the slope. Such interval ranged from 126 to 164 in water, from 38 to 155 in serum, and from 73 to 171 in urine. As such interval overlaps in all media assayed, it can be concluded that there are no significant matrix effects. The LOD and LOQ obtained met the clinically relevant range postsnakebite (from 0.01 to 100 μg/mL). The reference technique considered as the gold standard for snake venom toxin diagnosis is immunoassays. The only commercially available snakebite detection tool (considering it a traditional assay) is the Australian Commonwealth Serum Laboratories Snake Venom Detection Kit. Our approach presents several advantages over it since it can be applied to any type of venom (the traditional one is only available for specific Australian snakes) and does not suffer from cross-reactivity and low sensitivity.^8^ The LODs obtained with the MIPs MMs are like those obtained in LFA and paper-based optical assays for the determination of similar toxins.^13−18^ Also, the LODs are within the range of a multiplexed fluorescence assay (1 mg/mL).^21^ Remarkably, our moving-based assay has the lowest detection time reported (20 s) as compared with the 2 to 15 min required by the previously mentioned approaches. Portable detection can be achieved using a tailored smartphone with an integrated algorithm for signal processing and even in microplate readers, proving the feasibility of the approach for real practical applications.

**Figure 5:**
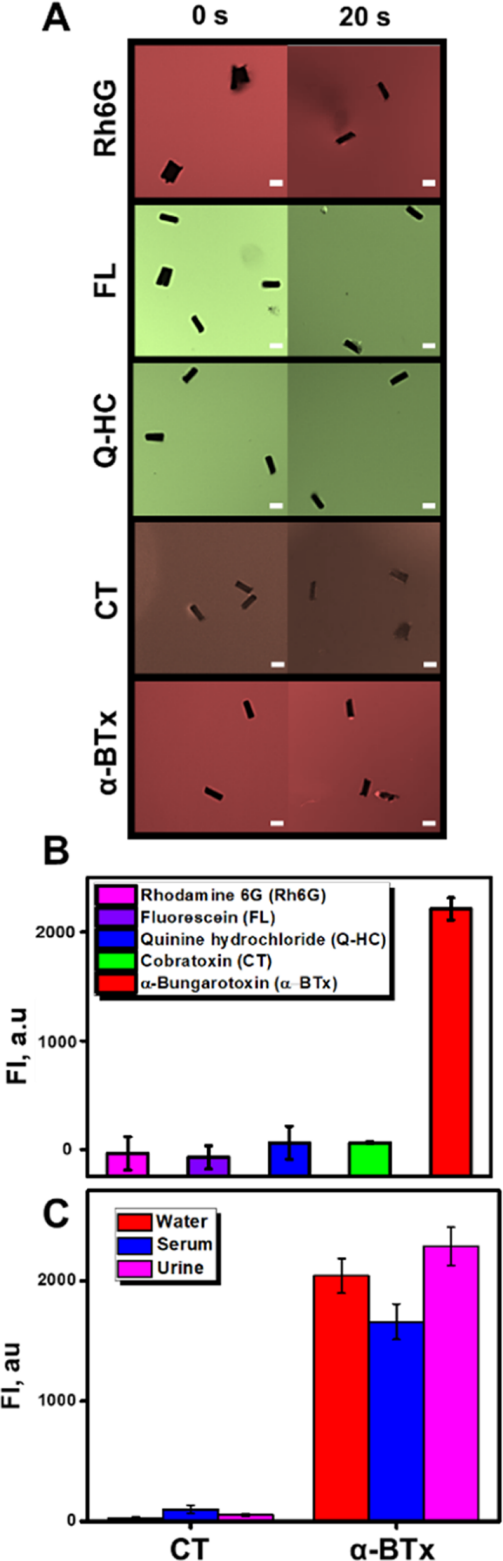
Selectivity of the MIP-MM moving sensor for α-BTx -TRITC detection. (A) Time-lapse fluorescence microscopy images and (B) corresponding FI plots in the presence of different interferences. Fluorescence values were plotted by subtracting the fluorescence values at time 0 from those at time 20‘’. Conditions: 3.3% PEG, 10% H_2_O_2_, response time, 20 s. Exposition time = 100 ms. Scale bars, 10 μm. *n* = 12. (C) Selectivity of the MIP-MM moving sensor for α-BTx-TRITC and α-cobratoxin-FITC simultaneously in water, serum, and urine. Corresponding FI plots were in the presence of 15 μg/mL α-BTx-TRITC with the filter G-2A and of 15 μg/mL α-cobratoxin-FITC with the filter C-FL-C FITC. Fluorescence values were plotted by subtracting the fluorescence values at time 0 from those at time 20 s. Conditions: 3.3% PEG, 10% H_2_O_2_, response time, 20 s. Exposition time = 100 ms. *n* = 12.

The selectivity of the protocol was tested among other small fluorescence molecules and a toxin with molecular weight and structure similar to those of our analyte (molecular weight, 8 kDa) (see Figure 5).

As can be seen in Figure 5A–C, no fluorescence increase in the MIP-MM surface (and hence, no interference) is noted after navigation in solutions containing small fluorescent molecules, namely, Rhodamine 6G (0.15 μg/mL, 0.47 kDa), fluorescein (60 μg/mL, 0.33 kDa), and quinine hydrochloride (60 μg/mL, 0.37 kDa). α-CT-FITC (15 μg/mL, 7.84 kDa) was also tested as interference. Such toxin can also be present in snake venom and the structure is like α-BTx-TRITC, with a single polypeptide chain of 62 amino acids, cross-linked by four disulfide bonds.^53^ As can be seen, no interference was noted, illustrating the high selectivity of our MMs. Additionally, we performed a selectivity experiment using solutions containing both α-BTx-TRITC and α-cobratoxin-FITC (15 μg/mL of both toxins) in water, urine, and serum. As can also be seen in Figure 5C, an increase in the fluorescence intensity on the MIP-MM surface was only noted for the α-BTx-TRITC in the samples, further revealing the high selectivity of our protocol even in the presence of similar molecules, thus holding considerable promise in future multiplexed assays.

**Figure 6:**
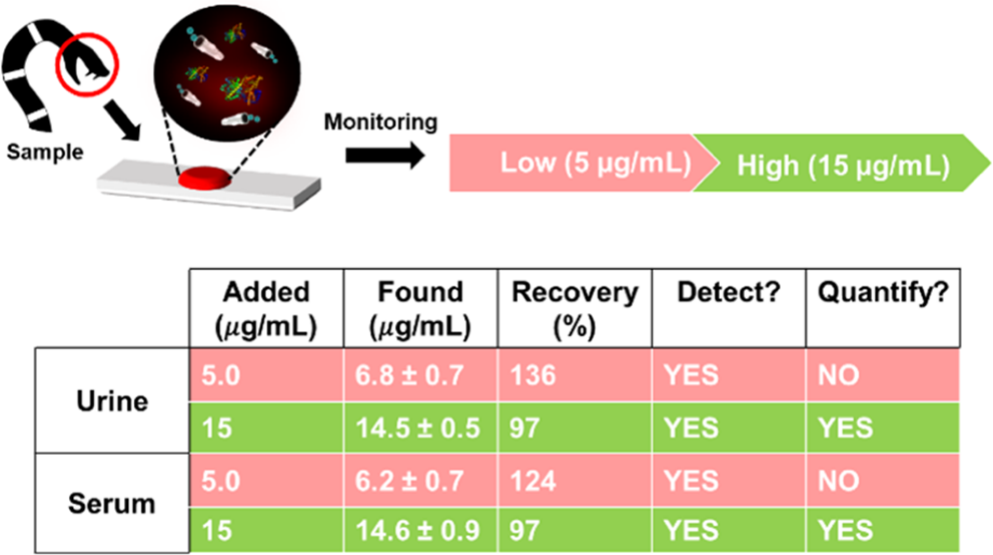
Performance of the MIP-MM moving sensor for α-BTx-TRITC detection in biological samples. Schematic of the assay for the fast monitoring (YES/NO detection) of α-BTx-TRITC in diluted urine and serum (top) and recovery results (bottom). Conditions: 3.3% PEG, 10% H_2_O_2_, response time, 20 s. *n* = 12.

Figure 6 illustrates a schematic of the practical applicability of the approach for the detection of a toxin in blood or urine. The sample just needs to be placed in a glass slide (even in the microscope or in a portable device), labeled, and after the addition of the MMs, tested for fluorescence. Recoveries were obtained by fortifying urine and serum samples at two levels (5 and 15 μg/mL). Good recoveries are obtained at the highest concentration level assayed, whereas slightly high values are noted for the lowest concentrations, reflecting the complexity of the samples. In all cases, the method can detect and quantify the target endotoxin with good precision and a relative standard deviation lower than 1%.

## Conclusions

We have demonstrated the applicability of MIP-MMs for the detection of snake venom toxins. For the first time, the full analytical potential of such ultraminiaturized tools has been fully characterized and demonstrated to the target application. The strategy relies on the specific recognition properties of the outer MM layer, as synthesized in the presence of the target toxin. The autonomous movement of the MIP-MMs results in the concentration of the target toxin on its surface after just 20 s of navigation, being one of the fastest MM-based sensing approaches developed to date. The limit of detection obtained, along with the high selectivity (even in the presence of toxins with similar structure), and the good performance in complex media analysis hold considerable promise for practical application. The versatility of the protocol allows for the further development of multiplexed and potential onsite assays using tailored MMs for specific analytes. While the detection is performed using the labeled analyte and a high-resolution optical microscope, other approaches, such as competitive assays (using labeled and nonlabeled analytes) and microplate readers, can be used for high-throughput and even smartphone-based portable detection for onsite fast diagnostics.

## Supporting Information Available

MIP-MMs propulsion at different H_2_O_2_ concentration (Video S1) (MP4)
MIP-MMs propulsion at different medium sample (Video S2) (MP4)
